# Isolated Idiopathic Aortitis in a Young Female Presenting With Exertional Chest Pain: A Case Report

**DOI:** 10.7759/cureus.63415

**Published:** 2024-06-28

**Authors:** Amit Raizada, Dhaval Trivedi

**Affiliations:** 1 Internal Medicine, New York Presbyterian Brooklyn Methodist Hospital, New York City, USA

**Keywords:** aortic valve insufficiency, vasculitis, young female, chest pain, idiopathic aortitis

## Abstract

Aortitis is a general term that describes inflammation of the aorta. In most cases, this inflammation is caused by an autoimmune etiology or an infectious etiology. In some instances, the underlying etiology may not be clear, and the diagnosis given is idiopathic aortitis. Cases of idiopathic aortitis are usually diagnosed based on histopathologic findings. Here, we present a case involving a 31-year-old female presenting with acutely worsening exertional shortness of breath and left-sided chest pain. An echocardiogram revealed a severely reduced ejection fraction with severe aortic regurgitation and diffusely increased aortic intima-media thickness. Bioprosthetic aortic valve replacement was performed with histology, showing findings consistent with aortitis, and the patient received the diagnosis of idiopathic aortitis. This case highlights the need to consider aortitis as a differential in young patients presenting with exertional chest pain and severe aortic insufficiency.

## Introduction

Aortitis is a general term describing inflammation of the aorta. Aortitis has a wide variety of causes and clinical presentations. The presentation can vary from asymptomatic incidental findings on histopathology to severe back, chest, or abdominal pain, as well as aortic insufficiency [1]. Broadly, the most common causes of aortitis can be grouped into two categories; infectious and rheumatologic [1]. Infectious aortitis most commonly results from *Salmonella *and *Staphylococcal *species. Tuberculous aortitis can be seen more frequently in developing countries, while syphilitic aortitis is more rarely seen. Rheumatologic aortitis can occur as a consequence of large-vessel vasculitides such as giant cell arteritis and Takayasu arteritis. Rheumatologic aortitis can also be seen in HLA-B27-associated spondyloarthropathies and sarcoidosis [1]. Some cases of aortitis have no clear infectious or non-infectious etiology. In these cases, the diagnosis given is isolated idiopathic aortitis [2]. Described below is a case of a 31-year-old female who presented with severe aortic insufficiency requiring aortic valve replacement, with subsequent biopsy reports showing isolated idiopathic aortitis. This case underscores the need to consider idiopathic aortitis in a young patient with exertional chest pain and aortic insufficiency.

## Case presentation

A 31-year-old female with a past medical history of uncontrolled type 2 diabetes mellitus, hidradenitis, anxiety, and intermittent palpitations presented with a one-week history of acutely worsening shortness of breath and left-sided chest pain that began shortly after starting CrossFit. Pain worsened with exertion and was relieved with rest, with no radiation. The patient denied numbness, tingling, or nausea. The patient was not currently on any home medications. The most recent hemoglobin A1c was 8.0%.

**Figure 1:**
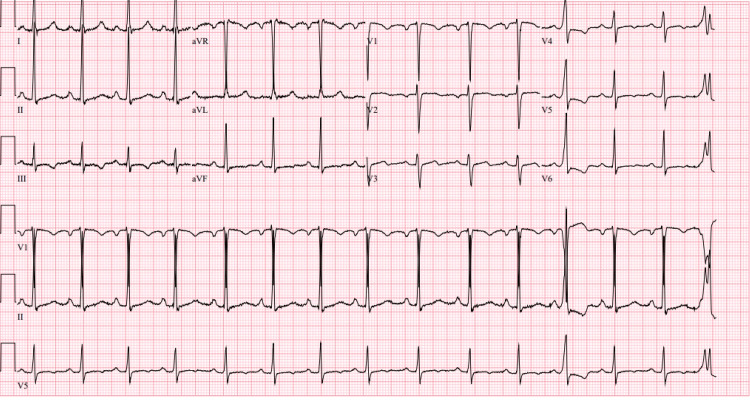
Electrocardiogram taken on admission showing a sinus rhythm with premature ventricular complexes

Vital signs were notable for blood pressure of 156/65 on the right arm and 157/68 on the left arm. On physical examination, a grade 2-3 systolic ejection murmur was heard. Distal pulses were noted to be normal and symmetric in bilateral upper and lower extremities. An electrocardiogram (EKG) on admission showed Sinus Rhythm with premature ventricular complexes (Figure 1). Labs showed an elevated pro-brain natriuretic peptide (pro-BNP) of 1637 and elevated troponins. A computed tomography (CT) of the chest with IV contrast was negative for pulmonary embolus. An echocardiogram was performed, showing an ejection fraction of 25-30% with severe global hypokinesis of the left ventricle and severe aortic regurgitation; however, the aortic valve was not well visualized. A stress echocardiogram was then performed one day later, showing severe aortic regurgitation, with a maximal heart rate of 169 beats per minute achieved. A stress electrocardiogram showed 1mm ST segment depressions in leads 1, V6, and the augmented vector foot (AVF) lead.

**Figure 2:**
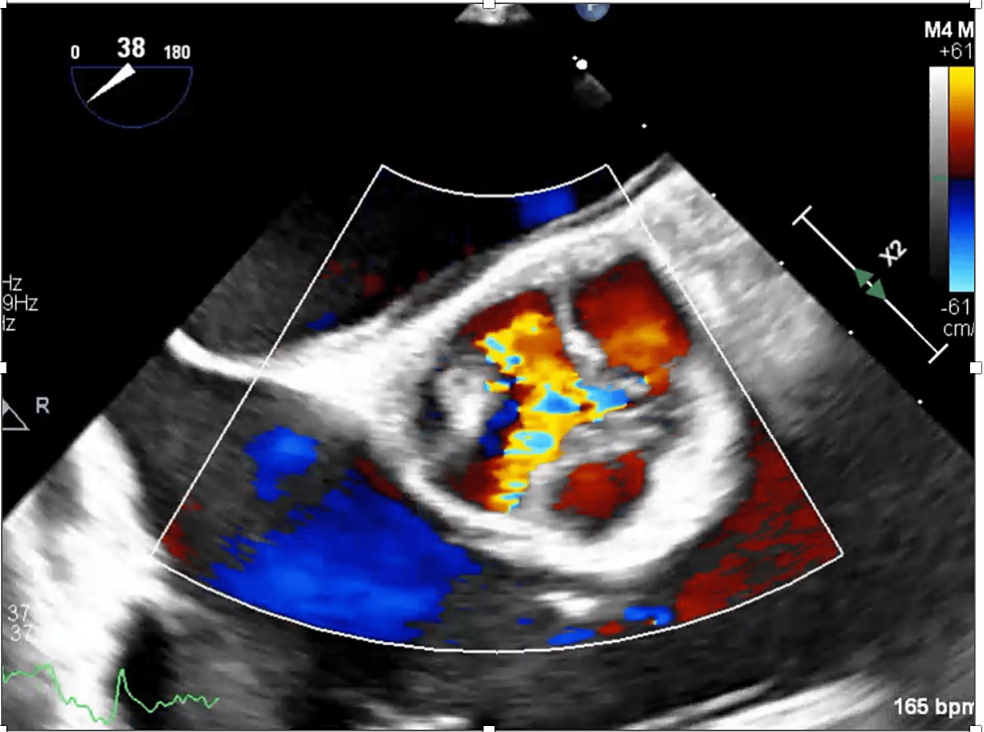
Color Doppler trans-esophageal echocardiogram showing severe aortic insufficiency

The patient subsequently underwent left heart catheterization and right heart catheterization one week after admission, showing severe aortic insufficiency, no angiographically significant coronary artery disease, as well as an elevated left ventricular end-diastolic pressure of 16 mmHg and a pulmonary capillary wedge pressure of 28. The patient was then started on intravenous furosemide and underwent a trans-esophageal echocardiogram (Figure 2) one day later, showing an ejection fraction of less than 20% with severe aortic regurgitation with possible non-coronary cusp prolapse, and diffusely increased aorta intima-media thickness. The ascending aorta is mildly dilated at 3.7 cm. The echocardiogram also showed a severely dilated left ventricle, trivial mitral regurgitation, trivial tricuspid regurgitation, and trivial pulmonic regurgitation.

**Figure 3:**
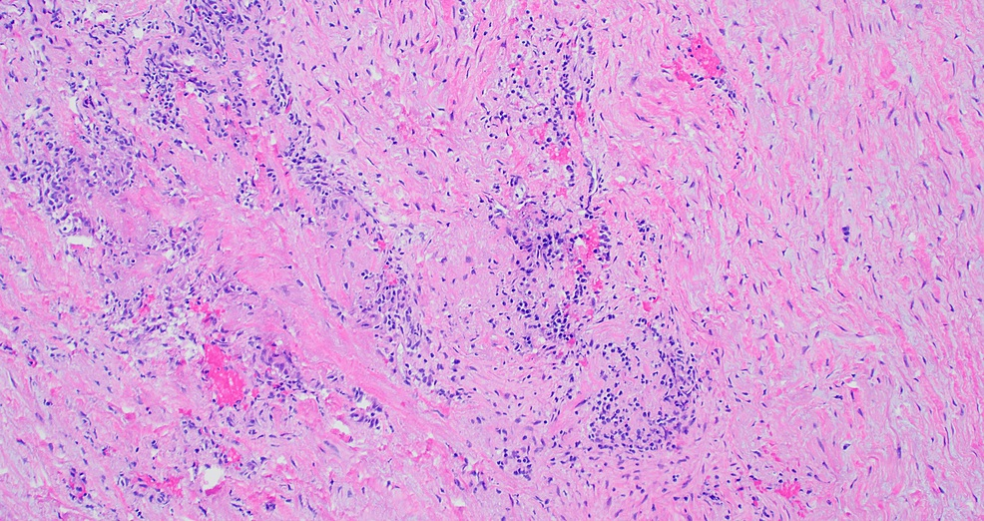
Histopathologic slide of aortic tunica media showing mononuclear cell infiltrate with no granuloma formation

A rheumatologic panel was sent, including anti-nuclear antibody (ANA), rheumatoid factor (RF), cyclic citrullinated peptide (CCP), erythrocyte sedimentation rate (ESR), C-reactive protein (CRP), and complement (Table 1). Infectious workup included testing for syphilis with *Treponema pallidum* test, and Quantiferon Gold to test for tuberculosis, both of which were negative. Rheumatologic workup was unrevealing, and the patient subsequently underwent bio-prosthetic aortic valve replacement one week later. Shown below are video clips of a trans-esophageal echocardiogram prior to surgery (Video 1) and a trans-thoracic echocardiogram performed three months after surgical intervention (Video 2) showing improvement in aortic insufficiency. Pathology slides such as the one shown below (Figure 3) were taken from a portion of the aortic wall, which showed mononuclear cellular infiltrates and myxoid degeneration of tunica media. There was no granulomatous inflammation or necrosis of giant cells seen in the sample.

**Table 1:** Selected laboratory values detailing a cardiac and rheumatologic workup pro-BNP - pro-brain natriuretic peptide; RF - rheumatoid factor; ANA - antinuclear antibody; CCP - cyclic citrullinated peptide; C3 - complement component 3; C4 - complement component 4; ESR - erythrocyte sedimentation rate; CRP - C-reactive protein

Test	Result	Reference range
Pro-BNP	1637 pg/ml	<= 125 pg/ml
Troponin 1	66.8 ng/L	0.0 – 53.7 ng/L
Troponin 2	45.9 ng/L	0.0 – 53.7 ng/L
RF	<10 IU/ml	0 – 14 IU/ml
ANA antibody	None detected	None detected
CCP antibody	3 units	0 – 19 units
C3 Complement	139 mg/dl	90 – 180 mg/dl
C4 Complement	37 mg/dl	10 – 40 mg/dl
ESR	15 mm/hr	0 – 30 mm/hr
CRP	<2.9 mg/L	<= 3.0 mg/L

**Video 1:** Video clip of trans-esophageal echocardiogram taken prior to surgery showing severe aortic insufficiency

**Video 2:** Trans-thoracic echocardiogram taken post-surgery showing significant improvement in aortic insufficiency

A repeat echocardiogram post-valve replacement showed an ejection fraction of 40-45%, with the bioprosthetic aortic valve in place with normal gradients and no significant regurgitation. The patient received the final diagnosis of idiopathic aortitis and has continued follow-up with rheumatology and cardiology as an outpatient. As part of the outpatient rheumatology follow-up, the patient underwent a positron emission tomography (PET) scan two months after surgery to check for signs of aortic inflammation, with the scan subsequently showing a negative result. The patient has been started on goal-directed medical therapy for the treatment of heart failure with reduced ejection fraction. 

## Discussion

Aortitis is a term that describes inflammation of the aortic wall [1]. Aortitis can be present in vasculitides such as giant cell arteritis and Takayasu arteritis, and can also be present in other autoimmune diseases such as systemic lupus erythematosus and rheumatoid arthritis. Aortitis can also occur because of infections, as seen in syphilis and tuberculosis, among other bacterial infections. Aortitis that is not attributable to any obvious infectious or non-infectious cause is termed isolated idiopathic aortitis [1].

The pathophysiology of aortitis differs based on the etiology. Generally, infectious causes of aortitis involve an atherosclerotic plaque or aneurysm sac in the wall of the aorta that becomes seeded by bacteria. With tuberculosis aortitis, seeding can occur from nearby infected tissues, such as lymph nodes. The infection leads to an inflammatory response that can lead to changes in the aortic wall, such as loss of elasticity, as seen in tuberculosis aortitis [1].

The non-infectious causes of aortitis most often involve granulomatous diseases such as giant cell arteritis and Takayasu arteritis, in which there is an inflammatory cellular infiltrate of the aortic wall with granuloma formation. Over time, this leads to scarring of the aortic media and can even lead to the destruction of the elastic lamina. Takayasu arteritis leads to intimal thickening and adventitial fibrosis, with subsequent luminal narrowing. Giant cell arteritis involves inflammation of the media and the formation of aortic aneurysms [1].

Cases of idiopathic isolated aortitis are usually diagnosed based on histopathologic findings, as there are no infectious or non-infectious causes that can be attributable to the aortitis [2]. These cases usually involve the ascending thoracic aorta. The histopathology can be like what is seen in other non-infectious causes of aortitis. There is an inflammatory infiltrate that usually involves the aortic media and changes consistent with chronic inflammation, including fibrosis [3]. However, in idiopathic aortitis, granuloma formation would not be seen. In the case of this patient, the diagnosis was only made upon histopathologic examination of the aortic valve, which showed a lymphocytic infiltrate with no granuloma formation.

Although cases of aortitis attributable to definitive infectious and non-infectious cases are well-reported, the prevalence of isolated idiopathic aortitis is not to be worth discussing as it is an under-recognized vasculitis. A study by Schmidt and colleagues reported on the prevalence and predictors of biopsy-proven aortitis in a 12-year nationwide Danish population study [3]. This study showed that of 1210 resected thoracic aorta samples, 74 (6.1%) were found to have aortitis. Almost three-quarters of those samples were idiopathic [3]. While this study did involve resected thoracic aorta specimens and, therefore, had a bias toward severe cases of aortitis requiring surgical resection, the results highlight that idiopathic aortitis is a vasculitis that is worth considering.

Management of aortitis is dependent on the etiology. Infectious aortitis is usually treated with a combination of surgical debridement and IV antibiotic therapy. For the most common causes of non-infectious aortitis - large-vessel vasculitides such as giant cell arteritis and Takayasu arteritis - treatment primarily involves corticosteroid therapy. Close monitoring is needed, however, as the rate of relapse can be 50% or greater after steroid therapy. For the management of isolated idiopathic aortitis, treatment largely depends on the extent of the disease. Patients can be managed with surgical intervention, as was seen by the patient described in this case study. Steroid therapy can also be considered as well, depending on the extent of the disease and the need for long-term therapy [4]. This patient was managed mainly with surgical valve replacement, followed by close cardiology and rheumatology follow-up as an outpatient.

## Conclusions

Aortitis should be considered as a differential diagnosis in a young patient presenting with left-sided exertional chest pain with no other cardiovascular risk factors. Patients with aortitis in which there is no clear infectious or inflammatory etiology are given the diagnosis of idiopathic aortitis and may require steroid therapy versus surgical intervention, depending on disease severity. In a case where aortitis is suspected with symptoms of chest pain, an echocardiogram must be obtained to assess the degree of aortic insufficiency. If significant aortic insufficiency is found, these patients require valve replacement to prevent further complications, including the development of congestive heart failure. Patients with idiopathic aortitis require close follow-up with cardiology and rheumatology.
